# A strategy to enhance and modify fatty acid synthesis in *Corynebacterium glutamicum* and *Escherichia coli*: overexpression of acyl-CoA thioesterases

**DOI:** 10.1186/s12934-023-02189-w

**Published:** 2023-09-21

**Authors:** Jin Liu, Mandlaa, Jia Wang, Ziyu Sun, Zhongjun Chen

**Affiliations:** Food Science and Engineering College, Inner Mongolia Agricultural University, 306 Zhaowood Road, Saihan District, Hohhot, 010018 Inner Mongolia China

**Keywords:** Fatty acid biosynthesis, Thioesterase, *Corynebacterium glutamicum*, *Escherichia coli*, Enzymatic characterization

## Abstract

**Background:**

Fatty acid (FA) is an important platform compound for the further synthesis of high‐value biofuels and oleochemicals, but chemical synthesis of FA has many limitations. One way to meet the future demand for FA could be to use microbial cell factories for FA biosynthesis.

**Results:**

Thioesterase (TE; TesA, TesB, and TE9) of *Corynebacterium glutamicum* (CG) can potentially improve FA biosynthesis, and *tesA*, *tesB*, and *te9* were overexpressed in *C. glutamicum* and *Escherichia coli* (EC), respectively, in this study. The results showed that the total fatty acid (TFA) production of CG*tesB* and EC*tesB* significantly increased to 180.52 mg/g dry cell weight (DCW) and 123.52 mg/g DCW, respectively (*P* < 0.05). Overexpression strains CG and EC could increase the production of C16:0, C18:1(t), C18:2, C20:1, C16:1, C18:0, and C18:1(c) (*P* < 0.05), respectively, and the changes of long-chain FA resulted in the enhancement of TFA production. The enzymatic properties of TesA, TesB, and TE9 in vitro were determined: they were specific for long-, broad and short-chain substrates, respectively; the optimal temperature was 30.0 °C and the optimal acid–base (pH) were 8.0, 8.0, and 9.0, respectively; they were inhibited by Fe^2+^, Cu^2+^, Zn^2+^, Mg^2+^, and K^+^.

**Conclusion:**

Overexpression TE enhances and modifies FA biosynthesis with multiple productive applications, and the enzyme properties provided useful clues for optimizing FA synthesis.

**Graphical Abstract:**

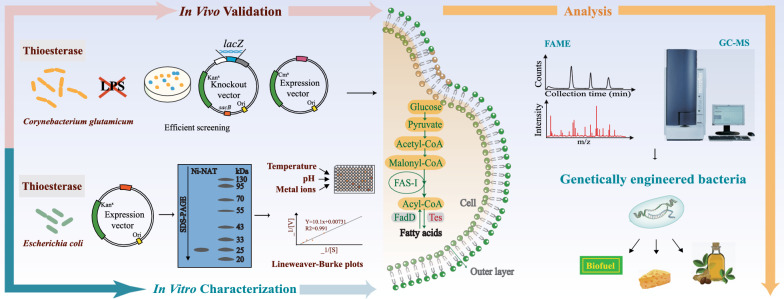

**Supplementary Information:**

The online version contains supplementary material available at 10.1186/s12934-023-02189-w.

## Background

Fatty acid (FA), an important platform compound in the production of biofuels, oleochemicals, fatty acid esters, and fatty alcohols, is widely used in the food, medical and chemical industry [1–3]. The processes used to prepare FA can be divided into chemical and biological synthesis. The complex reaction, poor product selectivity, and high cost of the chemical method have restricted its application [4]. However, FA biosynthesis is a promising alternative approach due to the mild synthesis condition, target product selectivity, and environmental sustainability of the process. Raw material as an important factor in the production of bio-based FA and its derivatives has changed over time [1]. The use of first-generation food crop feedstocks, second-generation raw and waste materials, and third-generation photosynthetic microbes (e.g., microalgae) has resulted in resource shortages and high production costs. Moreover, no natural producers have been isolated to produce industrially relevant quantities (~ 100 g/L titers) at high yields (> 90% of theoretical) [5]. The fourth generation of feedstocks, the genetic modification of microorganisms, is expected to solve those problems and achieve the sustainable production of FA and its derivatives by genetic engineering tools [6].

Model microorganisms (e.g., *Escherichia coli* and *Corynebacterium glutamicum*) are often used in engineering because of their well-established genetic background. *Escherichia coli* has been extensively studied due to its fast growth and amenability to genetic manipulation [7]. The genetic engineering of *E. coli* has enabled the high-titer production of FA and polyhydroxyalkanoates [2]. The fatty acid synthase (FAS) system is essential for FA de novo synthesis and *E. coli* contains a multienzyme mixture (FAS-II) whose metabolites are acyl-carrier protein (ACP) derivatives [8].

Unlike most bacteria, coryneform bacteria, such as *C. glutamicum*, contain multidomain complexes (FAS-I) with coenzyme A (CoA) derivatives as metabolites (Additional file 1: Fig. S1) [9]. There is no β-oxidation pathway for the FA degradation in *C. glutamicum* [9], which is conducive to the accumulation of free fatty acid (FFA). In addition, *C. glutamicum* is a Gram-positive bacterium that is non-pathogenic and does not produce potentially health-threatening endotoxins (i.e., lipopolysaccharide [LPS]). But, *E. coli,* a Gram-negative bacterium, contains LPS, which limits its applications in food and medicine. *C. glutamicum* has long been used for the industrial production of a variety of amino acids. Although the feasibility of FA production by *C. glutamicum* has been verified [10], there is the possibility of productivity improvement.

Thioesterase (TE) of *C. glutamicum* has the potential to be applied to enhance FA production [10]. TE cleaves acyl thioesters (CoA or ACP) to release FFA from biosynthetic pathways (Additional file 1: Fig. S1), and its substrate specificity dictates that it produces acyl-chain pools of a specific length, which are redoxed and further produce various derivatives. Thus, TE overexpression and increased TE activity promote FA production and can modify its composition [5]. It is extremely important to fully understand the characteristics of TE because practical applications are dependent on biochemical properties (e.g., substrate specificity, optimum temperature, and pH). Several families of acyl-CoA thioesterase (ACOT) have been identified in various species [10, 11]. Microbial TE often has a proofreading role in the cell and acts on more substrates than plant TE [12]. The TE activity of wild-type *C. glutamicum* (WG) is about 16-fold higher than that of *E. coli*. The *C. glutamicum* genome contains three putative ACOT genes: *tesA* (NCgl2365), *tesB* (NCgl1600), and *te9* (NCgl0090) [9]. TesA (GenBank accession number [GAN] WP004567531) encoded by *tesA* belongs to the TE1 family [10], and TesB (GAN WP011014521) encoded by *tesB* is a member of the TE4 family [13]. TE9 (GAN WP011013382) encoded by *te9* was annotated as a hydrolase or acyltransferase. However, their properties and functions have not been fully characterized.

In the present study, the putative TE genes *tesA*, *tesB*, and *te9* from *C. glutamicum* were homologously and heterologously overexpressed to improve FA production in the original strain, and the composition and changes of FA were investigated to obtain suitable engineered strains for various productive purposes. In addition, the enzymatic properties of the above three enzymes were studied in vitro to further corroborate and clarify their biological roles in homologous or heterologous overexpression, and to lay the foundation for further adjustments of the FA anabolic pathways.

## Materials and methods

### Strains, plasmids, and growth conditions

The strains, plasmids, and primers used in this work are given in Tables 1, 2, 3. Gene knockout and overexpression strains of *C. glutamicum* ATCC 13032 (GAN NC003450.3) were constructed by pk18*mobsacB* and pXMJ19, respectively. *E. coli* DH5α was used for DNA manipulation, and heterologous expression of genes was performed in *E. coli* BL21 (DE3) using pET-28a( +) and in *E. coli* DH5α using pXMJ19, respectively. FA production was performed by fermentation using engineered *C. glutamicum* as well as heterologous overexpressors of *E. coli* DH5α.

**Table 1:** Strains used in this study

Strain	Relevant genotype	Source/reference
*E. coli* K-12 MG1655	Wild-type strain	Miaoling Bio
BL21(DE3)	F^—^*ompT* hsd SB(r_B_^—^ m_B_^—^) gal dcm(DE3)	Angyubio
DH5*α*	F^—^φ80 *lacZ*ΔM15 Δ(*lacZYA*-*argF*)U169 *endA1 recA1 hsdR17* (r_K_^—^m_K_^+^) *supE44*λ^—^ *thi*-1 *gyrA96 relA1 phoA*	Angyubio
BL-1	Kan^R^; *E. coli* BL21(DE3)/pET-28a	This study
BL*tesA*	Kan^R^; *E. coli* BL21(DE3)/pET_*tesA*	This study
BL*tesB*	Kan^R^; *E. coli* BL21(DE3)/pET_*tesB*	This study
BL*te9*	Kan^R^; *E. coli* BL21(DE3)/pET_*te9*	This study
DHΔ*tesA*	Kan^R^; *E. coli* DH5α/pK18_*la*_*tesA*	This study
DHΔ*tesB*	Kan^R^; *E. coli* DH5α/pK18_*la*_*tesB*	This study
DH*te9*	Kan^R^; *E. coli* DH5α/pK18_*la*_* te9*	This study
DH*tesA*	Cm^R^; *E. coli* DH5α/ pX_*tesA*	This study
DH*tesB*	Cm^R^; *E. coli* DH5α/ pX_*tesB*	This study
DH*te9*	Cm^R^; *E. coli* DH5α/ pX_*te9*	This study
WG	Wild-type *C. glutamicum* ATCC 13032	Lab collection
CG*tesA*	Cm^R^; CGΔ*tesA*/pX_*tesA*	This study
CG*tesB*	Cm^R^; CGΔ*tesB*/pX_*tesB*	This study
CG*te9*	Cm^R^; CGΔ*te9*/pX_*te9*	This study
CGΔ*tesA*	*C. glutamicum* ATCC 13032 Δ*tesA*	This study
CGΔ*tesB*	*C. glutamicum* ATCC 13032 Δ*tesB*	This study
CGΔ*te9*	*C. glutamicum* ATCC 13032 Δ*te9*	This study

**Table 2:** Plasmids used in this study

Plasmid	Relevant genotype	Source
pK18*mobsacB*	Kan^R^, *sacB*; suicide plasmid for gene knockout in *C. glutamicum*	Lab collection
pET-28a	Kan^R^; plasmid for heterologous protein expression in *E. coli*, control-lable T7 promoter	Lab collection
pXMJ19	Cm^R^; *E. coli*–*C. glutamicum* shuttle plasmid for complementary overexpression in *C. glutamicum*, Tac promoter	Miaoling Bio
pET_*tesA*	Kan^R^; pET-28a carrying *tesA*	This study
pET_*tesB*	Kan^R^; pET-28a carrying *tesB*	This study
pET_*te9*	Kan^R^; pET-28a carrying *te9*	This study
pK18_*tesA*	Kan^R^; pK18*mobsacB* carrying the upper and lower homologous arms of *tesA*	This study
pK18_*tesB*	Kan^R^; pK18*mobsacB* carrying the upper and lower homologous arms of *tesB*	This study
pK18_*te9*	Kan^R^; pK18*mobsacB* carrying the upper and lower homologous arms of *te9*	This study
pK18_*la*_*tesA*	Kan^R^; pK18_*tesA* carrying *lacZ* (NC_000913)	This study
pK18_*la*_*tesB*	Kan^R^; pK18_*tesB* carrying *lacZ* (NC_000913)	This study
pK18_*la*_*te9*	Kan^R^; pK18_*te9* carrying *lacZ* (NC_000913)	This study
pX_*tesA*	Cm^R^; pXMJ19 carrying *tesA*	This study
pX_*tesB*	Cm^R^; pXMJ19 carrying *tesB*	This study
pX_*te9*	Cm^R^; pXMJ19 carrying *te9*	This study

The recombinant plasmids were used as follows: The pet_ series was used for thioesterase heterologous expression in *E. coli* BL21(DE3). The pK18_*la*_ series was used for gene knockout in *C. glutamicum*. The pX_ series was used for complementary overexpression in the knockout

**Table 3:** Primer sequences used in this study

Name	Forward primer (5′ → 3′)	Name	Reverse primer (5′ → 3′)
pE-5-f	GCGAACCATATGGCAGCCAACAATG	pE-5-r	AGCCGCGTCGACGGTGATAGAAAGAGTC
pE-6-f	GAGTGACATATGAAAACTATTGAAG	pE-6-r	TCCATGGTCGACGAGTTGGTAGTTGGAACTGA
pE-9-f	GCACGTCATATGTTTCTCACACTCT	pE-9-r	CACGCTGTCGACCCGCACTGAGGAGTTGATTA
pX-5-f	AAACAGAAGCTTAGAAAGAGGCTAG	pX-5-r	AGGCCAGAATTCCCTGAGGTGATAGAAAGAGT
pX-6-f	TCACCGCTGCAGATAAATTAATTGG	pX-6-r	CAGTATGAATTCAGGTCAAATGATGAAACTTA
pX-9-f	AGTACTTCTAGATGCAAATCTAGTA	pX-9-r	CAACCGCCCGGGGGAGTTGATTAACCTGCACG
pK-5-uf	CCAGCTGAATTCGCGCAATCTACGGTGGCA	pK-5-ur	AAGTAGACTAGTAGACTCTTTCTATCACCT
pK-5-df	ATGGGGACTAGTTAGCCTCTTTCTTTGTAG	pK-5-dr	GATTTTTCTAGAAGGGCTTTTTATCAGGAC
pK-6-uf	AACGCCGAATTCGCCGTATCCCTCGTA	pK-6-ur	TCACGCTCTAGAGTTCCTGCGTCCCT
pK-6-df	GGCAGCTCTAGAGCCACCGAAAGTCC	pK-6-dr	TTGAAACTGCAGAAGACCCTTCCAGATT
pK-9-uf	CAACGTGGATCCAAATGCTGTGCTGGAATA	pK-9-ur	AAGTACTCTAGAGCGTGGGCTGATTGTA
pK-9-df	CCTCCATCTAGAATTCTCCTGCGTCGTCT	pK-9-dr	CCACCTAAGCTTACGCCATTCTTCTCCC
*lac*-X-f	CCGTCTTCTAGATGAAAAGAAAAACCAC	*lac*-X-r	GCGAGATCTAGAAAATAGCGGCAAAAATAA
*lac*-S-f	CCGTCTACTAGTTGAAAAGAAAAACCAC	*lac*-S-r	GCGAGAACTAGTAAATAGCGGCAAAAATAA

Primer *lac*-X series amplified *lacZ* in pK18_*la*_*tesB* and pK18_*la*_*te9*. Primer *lac*-S series amplified *lacZ* in pK18_*la*_*tesA*. Genomic PCR verification of the knockouts was performed using the primer pK -uf and pK -dr series

*E. coli* and *C. glutamicum* were grown in Luria–Bertani (LB) media (10 g/L peptone, 5 g/L yeast extract, 5 g/L NaCl) at 37 °C at 200 rpm and 30 °C at 200 rpm, respectively, or LB agar plates (15 g/L agar powder based on LB medium). Flasks (250 mL) with 50 mL LB medium were used for fermentation. Protein induction was required for the fermentation process and in vitro characterization of the enzymes. The inducer Isopropyl-β-D-thiogalactopyranoside (IPTG) was added at a final concentration of 1 mM when OD_600 nm_ of the strains reached 0.6 and induction was carried out for 4 h [14]. *C. glutamicum* competent cells were prepared as described previously [14]. *E. coli* competent cells were purchased from Shanghai AngYuBio Biotech Co., Ltd. (Shanghai, China). Clones were screened with chloramphenicol (Cm) or kanamycin (Kan) (both 50 μg/mL for *E. coli* and 25 μg/mL for *C. glutamicum*). The chromogenic substrate 5-bromo-4-chloro-3-indolyl-D-galactopyranoside (X-gal, 40 μg/mL, [Solarbio, Shanghai, China]) was used for phenotypic screening. For selection against *sacB*-cassettes, salt-free LB plates containing 20% [w/v] sucrose (Suc, [Sigma, St. Louis, MO, USA]) were used. Considering that TesA-disrupted strains might show impaired growth, to restore growth and screen knockouts, LB medium was supplemented with 50 μg/mL sodium oleate and 50 μg/mL sodium palmitate, as well as 4 mg/mL MgSO_4_·7H_2_O to promote the dissolution of fatty acid salts [10].

### Recombinant DNA techniques

PrimeSTAR GXL DNA Polymerase, QuickCut Enzyme, Alkaline Phosphatase, and T4 DNA Ligase were purchased from Takara (Dalian, China). Bacterial genomic DNA was extracted using a TIANamp Bacteria DNA Kit (TIANGEN, Beijing, China). DNA was purified using the Zymoclean Gel DNA Recovery Kit (Zymo Research, Irvine, CA, USA). All positive clones were verified by PCR (Additional file 1: Figs. S2, S3, S4).

### Construction of E. coli overexpression strains

Overexpression strains of *E. coli* DH5α and *E. coli* BL21 were constructed using the IPTG-inducible vector pXMJ19 and pET-28a, respectively. Insert-vector ligation products were transferred into competent *E. coli* cells using standard protocols [9] and screened on resistance plates.

### Construction of knockout and overexpression strains of C. glutamicum

An efficient "blue spot selection" knockout method based on the blue-white screening principle was established in this study (Additional file 1: Fig. S5). *lacZ* encoding β-galactosidase was amplified from *E. coli* K-12. MG1655 genome (GAN NC000913.2) and inserted between the homologous arms of the target gene ligated into suicide vector pk18*mobsacB*. The recombinant plasmids were electrotransformed into *C. glutamicum* for an initial screen on LB/X-gal/Kan plates, followed by a second screen on LB/X-gal/Suc plates (Additional file 1: Fig. S6). Blue colonies were distributed one-to-one on LB/X-gal/Suc plates and LB/Kan plates using sterile toothpicks. Blue clones that grew on LB/X-gal/Suc plates but not on LB/Kan plates were sucrose resistant (Suc^r^) and kanamycin sensitive (Kan^s^) and were selected for validation. Compared with the traditional method [15], this method improved efficiency, and knockouts were usually available within 50 single colonies. Furthermore, the method was generalizable in bacterial knockouts as long as the strain itself did not contain *lacZ* and thus did not degrade X-gal. Overexpression vectors were introduced into the knockout and screened on LB/Cm plates.

### Analytical methods

FA was extracted from the target bacteria after fermentation and methyl-esterified according to previous methods [16]. Fatty acid methyl ester (FAME) was analyzed quantitatively by Gas chromatography–mass spectrometry (GC–MS, [Agilent 8890-7000D, CA, USA]) using an external standard method. The standard mixture (37 fatty acids from C4 to C24) was purchased from Sigma-Aldrich. The results were statistically evaluated using one-way ANOVA followed by Tukey’s multiple comparisons test or two-way ANOVA followed by Bonferroni’s multiple comparisons test. Comparisons were considered statistically significant if *P* < 0.05.

### Enzymatic characterization and assays

After IPTG induction, cells were resuspended by adding PBS and then lysed by ultrasonic disruption. Proteins were purified from the clear lysate (obtained after centrifugation at 10,000* g* for 30 min) using Ni–NTA resin (Qiagen, Germantown, MA, US) according to the manufacturer’s instructions. Protein concentration was determined by the Bradford method (Additional file 1: Fig. S7) [17]. Proteins were analyzed by sodium dodecyl sulfate polyacrylamide gel electrophoresis (SDS-PAGE).

Enzyme activity and substrate preference (C2–C20, [Sigma]) were studied at 30 °C with established methods [13]. The optimal conditions were determined at temperature ranging from 25 to 45 °C and pH ranging from 6.0 to 10.0. Relative activity was expressed as a percentage of the maximum (100%). The effect of metal ions i.e., Zn^2+^, Cu^2+^, Mn^2+^, Mg^2+^, K^+^, and Fe^2+^ was determined by incubating the enzyme samples with 1 mM of each metal ion under optimal conditions. Meanwhile, one control sample without having any metal ions was also included as blank (100%). The enzyme kinetics were determined by Lineweaver − Burk double reciprocal plot at the optimal substrate (Additional file 1: Fig. S8).

## Results and discussion

### FA synthesized by engineered C. glutamicum

**Fig. 1:**
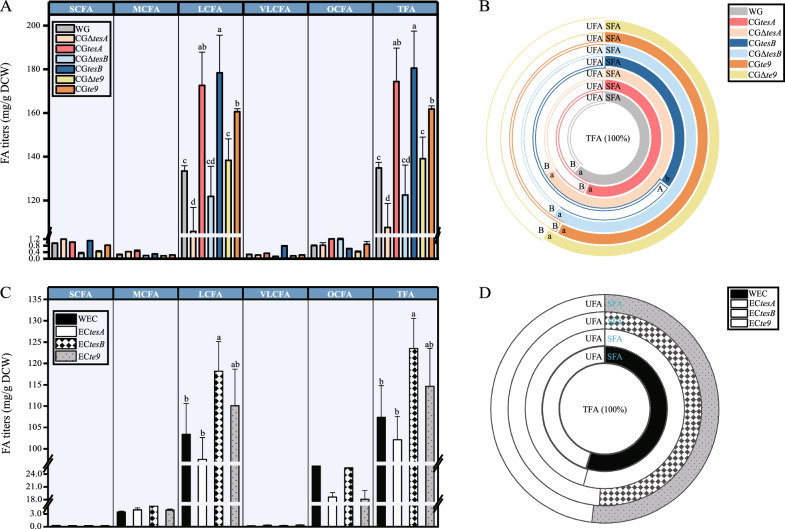
Changes of fatty acid types in engineered strains with the different expression levels of thioesterases. **A**, changes in titers of different fatty acid types of engineered *C. glutamicum*. **B**, concentric circle diagram reflecting the changes in the percentage of unsaturated and saturated fatty acids in engineered *C. glutamicum*. **C**, changes in titers of different fatty acid types of engineered *E. coli*. **D**, concentric circle diagram reflecting the changes in the percentage of unsaturated and saturated fatty acids in engineered *E. coli*. (*SCFA* short-chain fatty acid, *MCFA* medium-chain fatty acid; *LCFA* long-chain fatty acid, *VLCFA* very long-chain fatty acid, *OCFA* odd-chain fatty acid, *TFA* total fatty acid, *SFA* saturated fatty acid, *UFA* unsaturated fatty acid; *WG* wild-type *C. glutamicum*; CG*tesA*, CG*tesB*, and CG*te9*: *C. glutamicum* with overexpression of *tesA*, *tesB*, and *te9*, respectively; CGΔ*tesA*, CGΔ*tesB*, and CGΔ*te9*: *C. glutamicum* with *tesA*, *tesB*, and *te9* knocked out, respectively; WEC: wild-type *E. coli*; EC*tesA*, EC*tesB*, and EC*te9*: *E. coli* with overexpression of *tesA*, *tesB*, and *te9*). Different letters for the same fatty acid type indicated significant differences (*P* < 0.05; *n* = 3). Data without marked letters indicated a nonsignificant difference (ns)

**Fig. 2:**
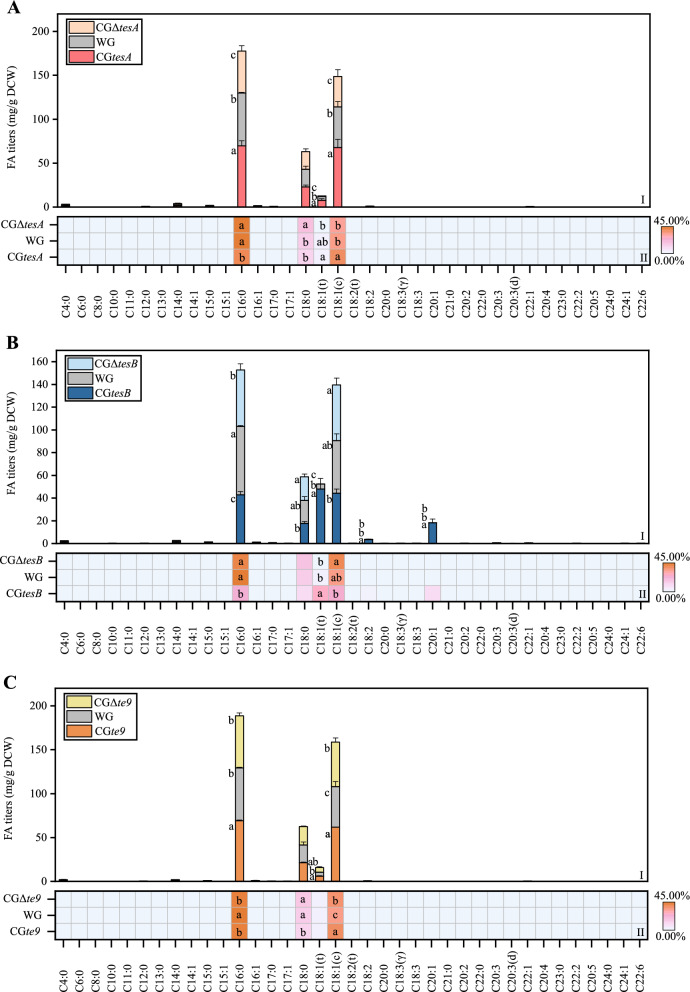
37 fatty acid production in engineered *C. glutamicum* with the different expression levels of thioesterases. **A**, **B** and **C**, 37 fatty acid production of engineered *C. glutamicum* strains with different expression levels of *tesA*, *tesB* and *te9*, respectively.(I) Stacked column chart demonstrating changes in 37 fatty acid titers of engineered *C. glutamicum* strains with the different expression levels of thioesterases. (II) Heatmap of percentage distribution (% of total fatty acid) of 37 fatty acids in engineered *C. glutamicum* strains with the different expression levels of thioesterases. (WG: wild-type *C. glutamicum*; CG*tesA*, CG*tesB*, and CG*te9*: *C. glutamicum* with overexpression of *tesA*, *tesB*, and *te9*, respectively; CGΔ*tesA*, CGΔ*tesB*, and CGΔ*te9*: *C. glutamicum* with *tesA*, *tesB*, and *te9* knocked out, respectively). Different letters for the same fatty acid indicated significant differences (*P* < 0.05; *n* = 3). Data without marked letters indicated a nonsignificant difference (ns)

To achieve controlled expression and reduce the feedback effects of the putative TE genes *tesA*, *tesB*, and *te9* in *C. glutamicum*, the three genes were homologously overexpressed in their respective knockout strain. The results (Fig. 1A) showed that total fatty acid (TFA) in the overexpression strain CG*tesA* was significantly increased by 29.35%, compared with WG, while that in the knockout strain CGΔ*tesA* was significantly reduced by 20.16% (*P* < 0.05). Both short-chain fatty acid (SCFA, < 6 carbon atoms) and medium-chain fatty acid (MCFA, 6 − 12 carbon atoms) were more abundant in CG*tesA* and CGΔ*tesA* than in the WG. The production of long-chain fatty acid (LCFA; 13 − 21 carbon atoms) and very long-chain fatty acid (VLCFA; > 22 carbon atoms) was promoted in CG*tesA* and conversely inhibited in CGΔ*tesA*. Significant changes in LCFA were responsible for the significant changes in TFA content in the *tesA* mutants. Moreover, the long-chain specificity of TesA was verified in vivo. The homologous overexpression of *tesA* also promoted the production of odd-chain fatty acid (OCFA) and unsaturated fatty acid (UFA) (Fig. 1A, B). Among the FA measured, the titers of palmitic acid (C16:0), elaidic acid (C18:1(t)), and oleic acid (C18:1(c)) were significantly increased in CG*tesA* by 15.96%, 78.23%, and 46.45%, respectively, but significantly decreased in CGΔ*tesA* compared with the WG levels (*P* < 0.05) (Fig. 2A). Therefore, TesA overexpression played a key role in C16:0, C18:1(t), and C18:1(c) synthesis in *C. glutamicum*. The percentage of C18:1(t) and C18:1(c) in CG*tesA* was increased compared with that in WG (Fig. 2AII). The stearic acid (C18:0) titers were not significantly affected by *tesA*, but the percentage of C18:0 in CGΔ*tesA* was significantly increased (*P* < 0.05). It has been reported that almost no FA was synthesized by the *tesA* knockout strain grown in minimal medium (MM) and the normal growth of the organism was prevented [10]. However, the FA production by CGΔ*tesA* grown in LB medium was significantly reduced but not completely lost (Fig. 1A), probably because MM could only satisfy the minimum nutritional requirements of the gene knockout strain, while LB medium was rich in various nutrients for CGΔ*tesA* growth.

Compared with WG, the TFA of the overexpression strain CG*tesB* was significantly increased by 33.88% (*P* < 0.05), while that of the knockout strain CGΔ*tesB* were reduced by 9.14% (Fig. 1A). SCFA and MCFA were increased in CG*tesB* and decreased in CGΔ*tesB*. LCFA and VLCFA increased by 33.70% (*P* < 0.05) and 201.79% in CG*tesB* while decreased by 8.66% and 41.11% in CGΔ*tesB*. The UFA was significantly increased (by 64.84%) in CG*tesB* (*P* < 0.05), but the synthesis of OCFA was inhibited (Fig. 1A, B). The titers of linoleic acid (C18:2), C18:1(t), and *cis*-11-eicosenoic acid (C20:1) were substantially increased in CG*tesB* compared with the WG (*P* < 0.05), and C18:1(t) was the predominant FA (Fig. 2B). The proportion of C18:2 and C20:1 increased in CG*tesB* (Fig. 2BII). In contrast, these fatty acids decreased to different extents in CGΔ*tesB*. Therefore, TesB overexpression played an important role in the synthesis of C18:1(t), C18:2, and C20:1. Unexpectedly, TesB overexpression had a negative effect on C18:0 production.

The TFA of the overexpression strain CG*te9* was significantly increased by 20.02% (*P* < 0.05) compared with WG. The TFA of the knockout strain CGΔ*te9* was slightly higher than that of the WG, possibly due to the regulatory effect of ACOT on intracellular acyl-CoA [18]. The short-chain preference of *te9* was verified by 54.50% reduction in the titer of SCFA in CGΔ*te9* compared with the WG and 92.33% increase in CG*te9* compared with CGΔ*te9*, albeit not to WG levels. In addition, the homologous overexpression of *te9* facilitated the production of OCFA and UFA. Similar to CG*tesA*, the expression levels of C16:0, C18:1(t), and C18:1(c) were significantly increased in CG*te9* (by 15.12%, 40.87%, and 33.30%, respectively) compared with the WG (*P* < 0.05) (Fig. 2CI). Except for C16:0, the percentage of C18:1(t) and C18:1(c) in CG*te9* increased to different degrees (Fig. 2CII).

Overexpression strains of *C. glutamicum* could be used to produce different types of FA. All these overexpression strains significantly promoted TFA production (*P* < 0.05) and were suitable for producing biofuels and high-value chemicals. Among them, CG*tesB* had the highest TFA content, i.e., 180.52 mg/g DCW. The carbon chain length and unsaturation of FA were altered in the overexpression strains. LCFA can be used in the treatment of several diseases [19], and the overexpression strains of *C. glutamicum* all significantly increased LCFA production, with CG*tesB* being the most prominent (*P* < 0.05). OCFA has recently attracted considerable interest due to its health benefits, and has been used as a platform compound to aid the production of biofuels and chemicals [6]. The superior capacity of *Y. lipolytica* to produce LCFA has been exploited to divert the metabolic flux of LCFA toward OCFA [20]. OCFA production could potentially be optimized by adjusting the metabolic fluxes of the overexpression strains. UFA is beneficial in terms of cold flow properties of biodiesel [21], and these overexpression strains promoted UFA synthesis, with CG*tesB* being the most prominent (*P* < 0.05). One possible explanation for how the overexpression of enzymes targets UFA is that the host cell membrane required UFA.

Overexpression strains could also be used to produce specific FA. Both CG*tesA* and CG*te9* significantly promoted the accumulation of C16:0, C18:1(c), and C18:1(t) (*P* < 0.05). These fatty acids can be used as eco-friendly biosurfactants for detergency, antimicrobial, and personal care applications, among others [22]. C16:0 and C18:1(c) are also used in the sustainable biodiesel industry [23]. C18:1(t) has a unique role in modulating hepatic lipogenesis [24]. CG*tesB* significantly increased the contents of C18:1(t), C18:2, and C20:1 (*P* < 0.05). In addition to the important function of C18:1(t), C18:2 has potential applications as biodiesel and biosurfactants, and for reducing inflammatory responses [22, 23]. C20:1 is an important component of biodiesel [23]. In conclusion, the engineered strains had a wide range of potential applications.

### FA synthesized by engineered E. coli

**Fig. 3:**
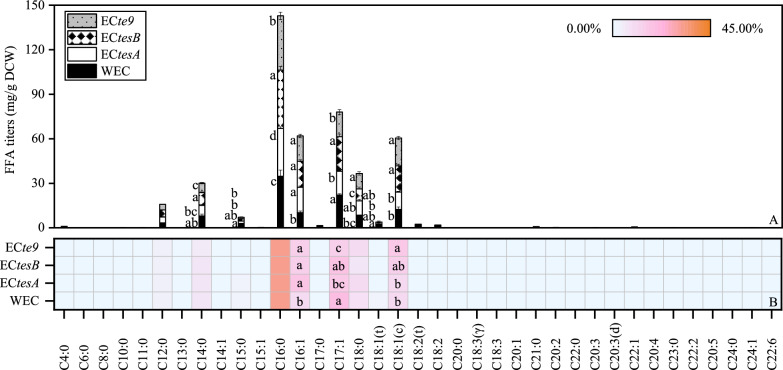
37 fatty acid production in engineered *E. coli* with overexpression of *tesA*, *tesB*, and *te9*. **A** Stacked column chart demonstrating changes in 37 fatty acid titers of engineered *E. coli* strains with overexpression of *tesA*, *tesB*, and *te9*. **B** Heatmap of percentage distribution (% of total fatty acid) of 37 fatty acids in engineered *E. coli* with overexpression of *tesA*, *tesB*, and *te9*. (WEC: wild-type *E. coli*; EC*tesA*, EC*tesB*, and EC*te9*: *E. coli* with overexpression of *tesA*, *tesB*, and *te9*). Different letters for the same fatty acid indicated significant differences (*P* < 0.05; *n* = 3). Data without marked letters indicated a nonsignificant difference (ns)

To improve the titer of FA in *E. coli* and improve the likelihood of obtaining engineered strains suitable for different productive uses, putative TE genes from *C. glutamicum* were heterologously overexpressed in *E. coli* DH5α. *E. coli* DH5α replicated stably and reduced the impact of gene modification, thus facilitating the expression of foreign genes [25]. The results showed that the FA content and composition were changed in the *E. coli* protein overexpression strain. The TFA content in the overexpression strain EC*tesA* was reduced by 4.93% compared with the wild-type *E. coli* (WEC) (Fig. 1C). The MCFA and VLCFA contents in EC*tesA* increased, with little change seen in SCFA, while the LCFA and OCFA contents decreased. The titer of palmitoleic acid (C16:1) in EC*tesA* was significantly increased (by 65.55%) compared with the WEC, while the titers of C16:0 and *cis*-10 heptadecenoic (C17:1) were significantly decreased (by 6.75% and 26.25%, respectively; *P* < 0.05) (Fig. 3A). The proportions of C16:1 and C17:1 also changed significantly (Fig. 3B). Therefore, EC*tesA* could be used for the sustainable production of C16:1.

The TFA content was significantly increased (by 15.02%; *P* < 0.05) in the overexpression strain EC*tesB* (Fig. 1C). The production of MCFA, LCFA, and VLCFA was substantially increased in EC*tesB* compared with the WEC, with a significant increase of 14.28% in LCFA (*P* < 0.05), while the production of SCFA and OCFA showed little change. In addition, the titers of C16:0, C16:1, and C18:1(c), which are functionally important, all increased significantly in EC*tesB* (by 12.64%, 71.80%, and 44.61%, respectively), although the levels of pentadecanoic acid (C15:0) and C18:1(t) were significantly decreased (*P* < 0.05) (Fig. 3A). The C16:1 proportion in EC*tesB* was also significantly higher than that in the WEC, and the percentage of C18:1(c) in EC*tesB* increased (Fig. 3B).

Compared with the WEC, the TFA content in engineered *E. coli* EC*te9* increased by 6.74% (Fig. 1C). Similar to EC*tesB*, the MCFA, LCFA, and VLCFA were increased in EC*te9*, and SCFA was almost unchanged. The OCFA content in EC*te9* was the lowest among the engineered *E. coli* strains, being 30.27% lower than in the WT. The results also showed that the titers of C16:0, C16:1, C18:0, and C18:1(c) in EC*te9* all increased significantly (by 5.69%, 66.78%, 21.14% and 52.60%, respectively), while those of myristic acid (C14:0), C15:0, and C17:1 decreased significantly (*P* < 0.05) (Fig. 3A). The percentage of C16:1 and C18:1(c) in EC*te9* was significantly increased compared with WEC (*P* < 0.05), and the C18:0 percentage in EC*te9* was increased (Fig. 3B). Although the titers and ratios of FA in these engineered *E. coli* strains differed considerably, the predominance of C16:0 did not change.

Thioesterase plays a key role in FA synthesis, which has led to the discovery of some promising TE genes. Engineered *E. coli* carrying TE genes from *Ricinus communis* and *Jatropha* spp. have been reported to accumulate > 2 g/L FFA [26]. Heterologous overexpression of TE (‘AcTesA) from *Acinetobacter baylyi* in *E. coli* boosted FA synthesis [27]. In this study, the content and composition of FA in the overexpression strains of *E. coli* were altered. Among them, EC*tesB* had the highest TFA content (123.52 mg/g DCW; *P* < 0.05) (Fig. 1C). EC*tesB* was also the most suitable strain for LCFA production. It has been suggested that the TE TesA from *Pseudomonas aeruginosa* may regulate the saturated/unsaturated FA ratio in membrane lipids [28]. The results of this study showed that the proportion of UFA was increased in all engineered *E. coli* (Fig. 1D). Thus, the three enzymes could preferentially act on specific unsaturated substrates in *C. glutamicum* and *E. coli*, resulting in increased production of UFA. All engineered *E. coli* strains also promoted C16:1 production. C16:1 has antioxidant properties and important applications in the manufacture of nut oils, cosmetics, and biodiesel [22]. In addition to C16:1, EC*tesB* favored the synthesis of C16:0 and C18:1(C), and EC*te9* promoted the accumulation of C16:0, C18:1(C), and C18:0. C18:0 also has important functions such as for biodiesel, biosurfactants, and reduction of inflammatory responses [22, 23].

Increased LCFA content was the main cause of increased TFA production. Overexpression of thioesterase also increased LCFA content when short-chain substrates, i.e., glucose or glycerol, were the sole carbon source (data not shown). All overexpression *C. glutamicum* strains had higher TFA levels than the overexpression *E. coli* strains. The SCFA, LCFA, and VLCFA titers were higher in the overexpression *C. glutamicum* strains compared with the overexpression *E. coli* strains, but this was not the case for MCFA or OCFA. The differences in FA content between the overexpression strains might be due to differences in the FA contents between their respective original strains. Furthermore, the enzymes tested in this study exhibited different substrate specificities in different organisms, resulting in different FA pools. For example, the overexpression of TesB and TE9, which promoted the production of SCFA in *C. glutamicum*, had little effect on SCFA in *E. coli*. TesA overexpression significantly promoted the synthesis of LCFA in *C. glutamicum* but adversely affected LCFA production in *E. coli*. Some studies have reported the production of FFA with different carbon lengths by the TE AcTesA in *E. coli* and *Synechocystis* [29]. The differences in FA content and composition might be related to their heterologous expression. The expression of the same protein in different organisms might affect the translation rate due to differences in the nature of the interaction between the amino acids and ribosomal exit tunnel in the new organism [30]. For heterologous and engineered enzymes, protein solubility may be an issue in vivo, which might also lead to differences [31]. In addition, there were differences in the guanine + cytosine content between *C. glutamicum* and *E. coli*, which might indirectly regulate and influence gene expression [32].

### Heterologous expression, purification, and characterization of enzymes

**Fig. 4:**
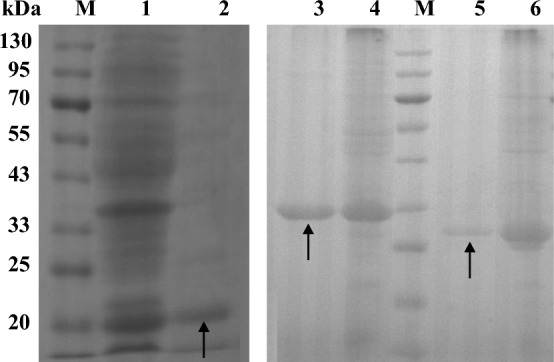
SDS-PAGE analysis of heterologous protein expression and purification. Lane M, prestained protein molecular weight marker; Lane 1, crude protein extracted from BL*tesA*; Lane 2, purified protein TesA; Lane 3, purified protein TesB; Lane 4, crude protein extracted from BL*tesB*; Lane 5, purified protein TE9; Lane 6, crude protein extracted from BL*te9*; Positions corresponding to TesA, TesB, and TE9 were indicated by arrows

Adding specific substrates and giving optimal conditions for key enzymes is an effective method to increase the amount of product in biosynthesis. In order to further explore the role of thioesterases in promoting FA synthesis, the characteristics of these enzymes were studied in vitro*.* The theoretical molecular weights of TesA, TesB, and TE9 registered in the National Center for Biotechnology Information (NCBI) database were 17.3, 31.7, and 27.9 kDa, respectively. SDS-PAGE analysis showed that the protein purified by Ni–NTA was relatively pure and approached the calculated value (Fig. 4).

**Fig. 5:**
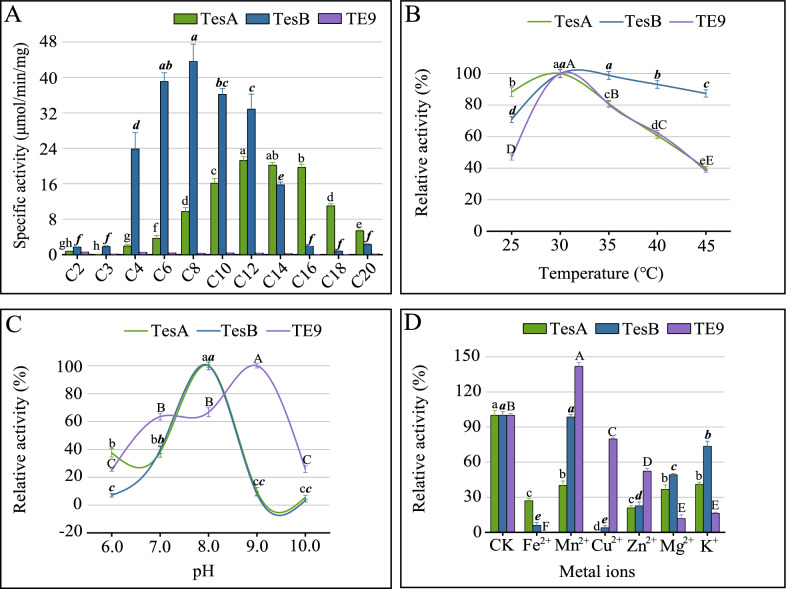
Characterization of thioesterases. **A**, thioesterase activities towards a range of acyl-CoA substrates. Effect of **B** temperature, **C** pH, and **D** metal ions on enzyme activities. CK, blank control without metal ions. The properties of TesB were derived from the results of our previous studies [35, 36]. Different letters for the same enzyme indicated significant differences (*P* < 0.05; *n* = 3). Data without marked letters indicated a nonsignificant difference (ns)

The substrate specificity of these three enzymes was verified in vitro. As shown in Fig. 5A, TesA had a preference for medium-long-chain substrates (C6 − C20; *P* < 0.05) and showed the highest activity with lauroyl-CoA (C12). This explained the increased production of MCFA in both overexpression strains, CG*tesA* and EC*tesA*, even though TesA was not shown to be medium-chain specific in *C. glutamicum*. In vitro validation might not be consistent with in vivo assays because enzyme activity might be affected by substrate availability and presentation in vivo [33]. The substrate specificity was influenced by the geometry and hydrophobicity of the catalytic pocket [34]. Furthermore, TesA from other organisms exhibited lysophospholipase A, protease, and arylesterase activities, in addition to TE activity [28]. The versatility of TesA from *C. glutamicum* needs to be determined and it might be worth considering whether TE activity could be enhanced by rational mutagenesis, such as the evolution of a few amino acid exchanges [27].

The substrate preference of TesB was characterized in vitro in the previous study [35, 36]. TesB was found to have broad substrate specificity and preferred C4−C14 carbon-length acyl-CoA, especially octanoyl-CoA (C8) (*P* < 0.05) (Fig. 5A). This was consistent with the broad chain length specificity of TesB demonstrated in vivo and was supported by the structural analysis of the active site [13]. The specificity of TesB determined in this study was identical to that of TesB from *Yersinia pestis* but differed from that of the long-chain-specific *E. coli* TesB and *Pseudomonas putida* TesB [37]. Consistent with the negative effect of TesB on C18:0 production in *C. glutamicum* (Fig. 2B), TesB had a low preference for stearoyl-CoA (C18) in vitro (data not shown).

The product encoded by *te9*, which has not previously been reported, has been annotated as a soluble protein possibly involved in FA synthesis and phospholipid homeostasis. Among all substrates tested, the activity for TE9 was relatively low; moreover, short-chain specificity was seen, especially for acetyl-CoA (C2) (Fig. 5A). This was consistent with the short-chain preference of TE9 demonstrated in vivo. The ACOT from *Bacillus halodurans* was also short-chain specific, and structural characterization revealed an internal channel at the active site that could accommodate SCFA; this may be a fatty acyl binding pocket [37]. This study showed that TesA, TesB, and TE9, which had different substrate specificities, are biologically active and able to hydrolyze acyl-CoA thioesters.

Some studies have reported that TesB from *E. coli* showed substrate ambiguity and activity toward the precursor 3-hydroxyvaleryl-CoA [11]. Substrate ambiguity is an inherent feature of the TE of the hotdog fold superfamily, which provides benefits to the cell such as proofreading and balancing of metabolite pools [18]. The TE in this study acted on the FA synthesis precursors C2 and malonyl-CoA (C3), which exhibited substrate ambiguity (although their structures were unknown). Compared with the precursors, TesA and TesB exhibited greater activity toward the downstream acyl-CoA, which reduced upstream acyl-CoA depletion. Thioesterases with different substrate specificities were chimerized to control the alkyl chain length [38]. Structural analyses could explain and allow for the optimization of TE functions; for example, protein hybrids might improve TE activity and substrate specificity.

Temperature affects bacterial growth and enzymatic reactions in cellular metabolism. The results showed that all three enzymes had the highest activity at 30 ℃ (Fig. 5B) [36], which is the optimum growth temperature of *C. glutamicum* [14]; this was probably because the enzyme activity depended mostly on the growth temperature of the enzyme-producing bacteria. The TE activities began to decrease significantly as the temperature increased (*P* < 0.05), similar to TE Them1; this was probably because the high temperature changed the enzyme’s spatial structure [39]. As shown in Fig. 5C, TE activities were altered significantly with increasing pH (*P* < 0.05). Changes in pH influence the ionization state of active site amino acids, and either enhance and stabilize interactions with the substrate or break intra- and intermolecular bonds [40]. As with a TE from a hot spring [34], TesA and TesB had the highest activity at pH 8.0 (Fig. 5C) [36]. TE9 operated over a wide pH range, with optimal activity seen at pH 9.0.

Metal ions have a stabilizing effect on enzymes and are necessary to maintain enzyme structure, enhance enzyme activity, and protect it from thermal inactivation [17]. Thioesterases are not metalloenzymes, and α-carbonic anhydrase (EC 4.2.1.1) is the only TE that acts via the metal hydroxide mechanism [41]. No studies of the effect of metal ions on the TE from *C. glutamicum* have been published. As shown in Fig. 5D, all ions significantly inhibited TesA activity (*P* < 0.05). Fe^2+^ and Cu^2+^ strongly inhibited TesB activity (*P* < 0.05); Mn^2+^ had less effect [36]. Surprisingly, 1 mM Mn^2+^ significantly promoted TE9 activity, with a maximum value of 141.84% (*P* < 0.05); therefore, TE9 was likely to be Mn^2+^-dependent. Moreover, Fe^2+^ and Cu^2+^ eliminated TE9 and TesA activity, respectively, probably because metal ions displaced other ions in the enzyme catalytic sites due to their similar chemical coordination. The Mg^2+^ ion inhibited TE activities in brown adipose tissue mitochondria and *Lactococcus lactis* [42]. The three enzymes of *C. glutamicum* were also inhibited by Mg^2+^ in this study, especially TE9 activity was reduced to 11.76% (*P* < 0.05) (Fig. 5D). Zn^2+^ may inhibit the activity of TE [43]. This study confirmed that Zn^2+^ inhibited the activity of the aforementioned enzymes (*P* < 0.05), possibly through competitive inhibition by binding to the enzymatic active sites. The active catalytic domains of these enzymes might contain potential metal ion binding sites.

The maximum reaction speed (*V*_max_) and Michaelis constant (*K*_M_) of TesA estimated through fitting to the Michaelis–Menten equation (*R*^*2*^ = *0.991*) were 136.79 μmol/L/min and 1.38 mM, respectively. This indicated that TesA was tightly bound to the lauroyl-CoA substrate. The Michaelis–Menten equation (*R*^*2*^ = *0.997*) demonstrated that the *V*_max_ and *K*_M_ of TesB were 558.65 μmol/L/min and 5.45 mM, respectively; this high *V*_max_ value might indicate an efficient reaction of TesB with octanoyl-CoA or a rapid reaction rate. TE9 (*V*_max_ = 7.35 μmol/L/min, *K*_M_ = 1.81 mM) had a lower catalytic level but higher substrate affinity for acetyl-CoA, as determined by the Michaelis–Menten equation (*R*^*2*^ = *0.992*). It was speculated as to why this organism had naturally high TE activity. Unlike *E. coli*, *C. glutamicum* has a unique lipid homeostasis mechanism [10], namely, a futile cycle mediated by ACOT (e.g., Tes) and acyl-CoA synthetase (e.g., FadD) (Additional file 1: Fig. S1), and it enhanced FA production by disrupting *fadD* and amplifying Tes. The coupling of Tes and FadD provided FFA for the synthesis of the mycolic acid layer and recycled the excess for membrane lipid synthesis. In addition, ACOTs might have START structural domains and their mechanism for optimal TE activity might be different [39]. This study also supported the hypothesis that FA production can be increased by enhancing the expression of more catalytically active TE. The in vitro characterization of the enzymatic properties performed in this study further clarified the biological role of these enzymes in vivo and provided a basis for optimizing FA biosynthesis. (Additional file 2).

## Conclusion

Overexpression of *tesA*, *tesB*, and *te9* in *C. glutamicum* (CG) and *E. coli* (EC), with CG*tesB* and EC*tesB* showing the highest TFA content (i.e., 180.52 mg/g DCW and 123.52 mg/g DCW, respectively,* P* < 0.05). The titers of C16:0, C16:1, C18:0, C18:1, C18:2, and C20:1 were significantly increased in the overexpression strains, and changes in LCFA resulted in the enhancement of TFA production. Moreover, TesA, TesB, and TE9 were long-, broad, and short-chain specific in vitro, inhibited by Fe^2+^, Cu^2+^, Zn^2+^, Mg^2+^, and K^+^, and being highest activity 30.0 °C and pH 8.0, 8.0, and 9.0, respectively. Overexpression strains could be used for various productive purposes and the enzyme properties provided useful clues for optimizing FA synthesis.

### Supplementary Information

**Additional file 1: Fig. S1.** Lipid metabolism and the predicted regulatory mechanisms in *Corynebacterium* *glutamicum*. There was a type I fatty acid synthase (FAS-I) system in *C* *glutamicum*, and the metabolites were CoA derivatives. As a TetR-type transcriptional regulator, FasR affected the transcriptional expression of genes including accD1, fasA, and fasB. Meanwhile, acyl-CoA could inhibit Acc, FasA, and FasB. This organism did not degrade fatty acid naturally due to the lack of the β-oxidation pathway. The red lines represented reference to previous studies (Ikeda et al. 2020), where double lines indicated inhibition and predicted inhibition and solid and dashed arrows indicated single and multiple enzymatic processes, respectively. The blue lines showed the predicted reverse β-oxidation pathway, a novel fatty acid synthesis pathway. Acetyl CoA carboxylase (Acc) was composed of AccBC, AccD1, and AccE. AccBC, acetyl-CoA carboxylase α subunit; AccD1, acetyl-CoA carboxylase β subunit; AccE, acyl carboxylase ε subunit; NCgl2309, acetyl-CoA acetyltransferase; NCgl0919, enoyl-CoA hydrolase; NCgl0973, acyl-CoA dehydrogenase; PD, pyruvate dehydrogenase; FasA, fatty acid synthase IA; FasB, fatty acid synthase IB; FasR, fatty acid synthesis inhibitory protein; Tes, acyl-CoA thioesterase; FadD, Acyl-CoA synthase. The dotted boxes in the figure were exogenous genes. FadE and Ter, acyl-CoA dehydrogenase; EchA, enoyl-CoA hydratase; FadB, multifunctional enoyl-CoA hydratase, 3-hydroxyacyl-CoA dehydrogenase; FadA, ketoacyl-CoA reductase. **Fig S2.** Agarose gel electrophoresis of the positive clones for heterologous expression in E. coli. **A** PCR validation of bacterial liquid. Lane M_2_, 2000 bp DNA marker; Lane b_A_, bacterial liquid PCR of BLtesA; Lane b_B_, bacterial liquid PCR of BLtesB; Lane b_9_, bacterial liquid PCR of BLte9; **B** double digestion validation of the recombinant plasmid. Lane M_5_, 5000 bp DNA marker; Lane d_A_, double digestion of plasmid pET_tesA extracted from BLtesA; Lane d_B_, double digestion of plasmid pET_tesB extracted from BLtesB; Lane d_9_, double digestion of plasmid pET_te9 extracted from BLte9. **Fig S3.** Agarose gel electrophoresis showed the PCR products of genomic DNA extracted from the knockouts. Lane M_5_, 5000 bp DNA marker; Lane −, negative control; Lane +, positive control; Lane a_A_ and b_A_, PCR products of genomic DNA from CGΔtesA; Lane v_A_, PCR product of pK18_tesA without lacZ; Lane a_B_, b_B_ and c_B_, PCR products of genomic DNA from CGΔtesB; Lane v_B_, PCR product of pK18_tesB without lacZ; Lane a_9_, b_9_ and c_9_, PCR products of genomic DNA from CGΔte9. Non-specific amplification in Lane a_9_ probably due to impure colonies, mixed with misassembled clones; Lane G, PCR product of genomic DNA from C. glutamicum. **Fig. S4.** Agarose gel electrophoresis of the positive complementations. **A** PCR validation of bacterial liquid. Lane M_2_, 2000 bp DNA marker; Lane h_A_, bacterial liquid PCR of CGtesA; Lane h_B_, bacterial liquid PCR of CGtesB; Lane h_9_, bacterial liquid PCR of CGte9; **B** double digestion validation of the recombinant plasmid. Lane M_5_, 5000 bp DNA marker; Lane q_A_, double digestion of plasmid pX_tesA extracted from CGtesA; Lane q_B_, double digestion of plasmid pX_tesB extracted from CGtesB; Lane q_9_, double digestion of plasmid pX_te9 extracted from CGte9. **Fig. S5.** Schematic diagram of gene knockout. **A** An efficient ‘‘blue spot selection’’ knockout method. LB, Luria-Bertani media; X-gal, 5-bromo-4-chloro-3-indolyl-D-galactopyranoside; Kan, kanamycin; Suc, sucrose. **B** Recombinant knockout vectors.** Fig. S6** Colony morphology. **A** C *glutamicum* wild type grown on LB/X-gal plate. **B** *C* *glutamicum* electrotransformants grown on LB/X-gal/Kan plate, blue colonies might be positive clones carrying recombinant knockout vectors. **Fig. S7.** Standard curve of BSA. (OD: Optical Density; BSA: Bovine Serum Albumin). **Fig. S8.** Lineweaver-Burke plots of TesA **A**, TesB **B**, and TE9 **C**.

**Additional file 2:** Minimal data set underlying the results described in this paper.

## Data Availability

The datasets supporting the conclusions of this article are included within the article and its additional files.

## Abbreviations

FA: Fatty acid
TE: Thioesterase
FFA: Free fatty acid
FAME: Fatty acid methyl ester
FAS: Fatty acid synthase
ACP: Acyl-carrier protein
ACOT: Acyl-CoA thioesterase
GAN: GenBank accession number
NCBI: National Center for Biotechnology Information
X-gal: 5-Bromo-4-chloro-3-indolyl-D-galactopyranoside
IPTG: Isopropyl-β-D-thiogalactopyranoside
GC–MS: Gas chromatography–mass spectrometry
SDS-PAGE: Sodium dodecyl sulfate polyacrylamide gel electrophoresis
TFA: Total fatty acid
SCFA: Short-chain fatty acid
MCFA: Medium-chain fatty acid
LCFA: Long-chain fatty acid
VLCFA: Very long-chain fatty acid
OCFA: Odd-chain fatty acid
UFA: Unsaturated fatty acid
C14:0: Myristic acid
C15:0: Pentadecanoic acid
C16:0: Palmitic acid
C16:1: Palmitoleic acid
C17:1: *Cis*-10 heptadecenoic
C18:0: Stearic acid
C18:1(T): Elaidic acid
C18:1(c): Oleic acid
C18:2: Linoleic acid
C20:1: *Cis*-11-Eicosenoic acid
C8: Octanoyl-CoA
C18: Stearoyl-CoA
C12: Lauroyl-CoA
C2: Acetyl-CoA
C3: Malonyl-CoA
*V*_max_: Maximum reaction speed
*K*_M_: Michaelis constant
