# Supplementary figures and images for: A strategy to enhance and modify fatty acid synthesis in Corynebacterium glutamicum and Escherichia coli: overexpression of acyl-CoA thioesterases
Source: Microb Cell Fact. 2023 Sep 21;22:191. doi: 10.1186/s12934-023-02189-w (PMC10512533; doi:10.1186/s12934-023-02189-w)

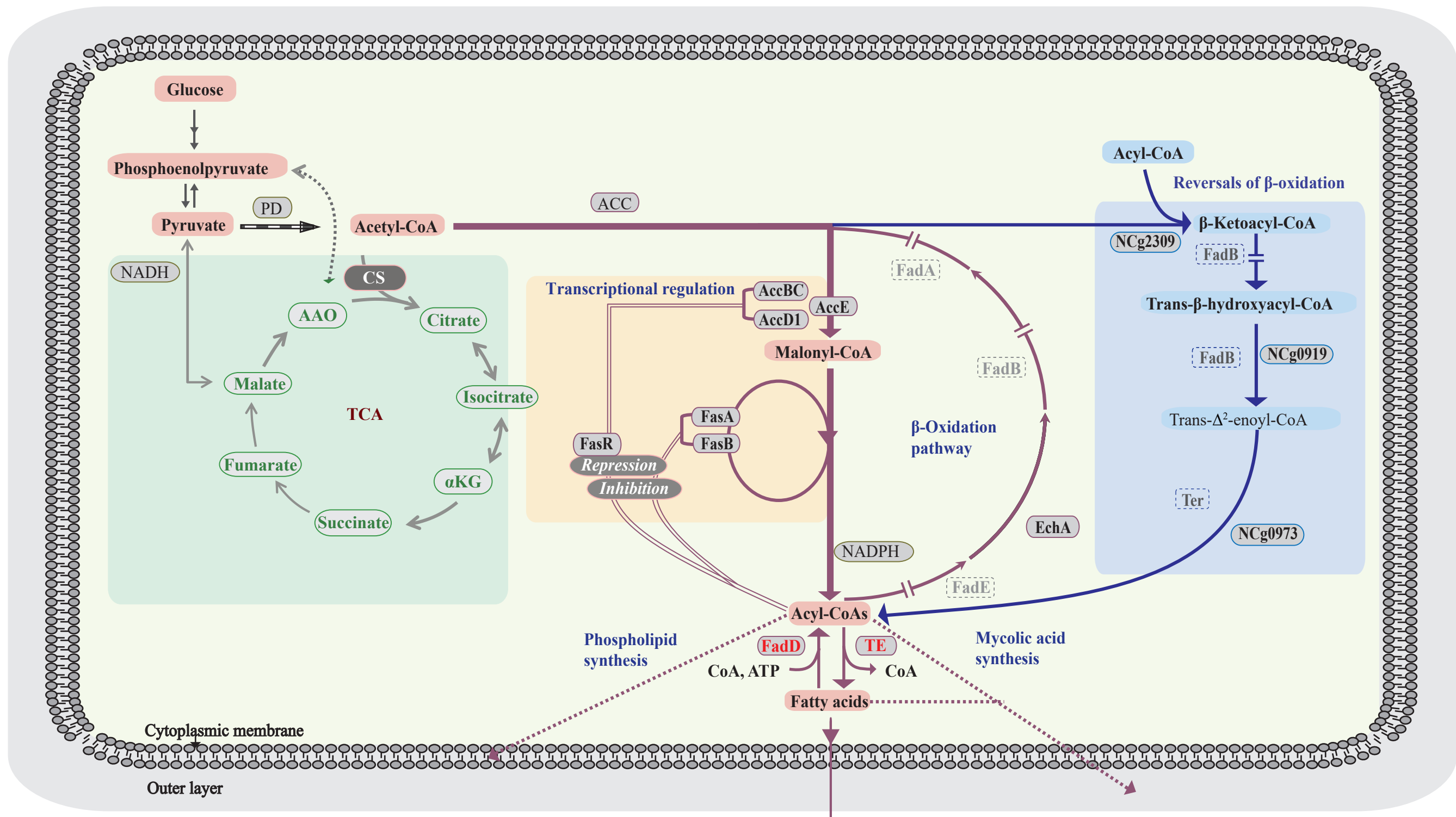

Supplement: Supplementary file 1 — Additional file 1: Fig. S1. Lipid metabolism and the predicted regulatory mechanisms in Corynebacterium glutamicum. There was a type I fatty acid synthase (FAS-I) system in C glutamicum, and the metabolites were CoA derivatives. As a TetR-type transcriptional regulator, FasR affected the transcriptional expression of genes including accD1, fasA, and fasB. Meanwhile, acyl-CoA could inhibit Acc, FasA, and FasB. This organism did not degrade fatty acid naturally due to the lack of the β-oxidation pathway. The red lines represented reference to previous studies (Ikeda et al. 2020), where double lines indicated inhibition and predicted inhibition and solid and dashed arrows indicated single and multiple enzymatic processes, respectively. The blue lines showed the predicted reverse β-oxidation pathway, a novel fatty acid synthesis pathway. Acetyl CoA carboxylase (Acc) was composed of AccBC, AccD1, and AccE. AccBC, acetyl-CoA carboxylase α subunit; AccD1, acetyl-CoA carboxylase β subunit; AccE, acyl carboxylase ε subunit; NCgl2309, acetyl-CoA acetyltransferase; NCgl0919, enoyl-CoA hydrolase; NCgl0973, acyl-CoA dehydrogenase; PD, pyruvate dehydrogenase; FasA, fatty acid synthase IA; FasB, fatty acid synthase IB; FasR, fatty acid synthesis inhibitory protein; Tes, acyl-CoA thioesterase; FadD, Acyl-CoA synthase. The dotted boxes in the figure were exogenous genes. FadE and Ter, acyl-CoA dehydrogenase; EchA, enoyl-CoA hydratase; FadB, multifunctional enoyl-CoA hydratase, 3-hydroxyacyl-CoA dehydrogenase; FadA, ketoacyl-CoA reductase. Fig S2. Agarose gel electrophoresis of the positive clones for heterologous expression in E. coli. A PCR validation of bacterial liquid. Lane M2, 2000 bp DNA marker; Lane bA, bacterial liquid PCR of BLtesA; Lane bB, bacterial liquid PCR of BLtesB; Lane b9, bacterial liquid PCR of BLte9; B double digestion validation of the recombinant plasmid. Lane M5, 5000 bp DNA marker; Lane dA, double digestion of plasmid pET_tesA extracted from BLtesA; Lane dB, [file 12934_2023_2189_MOESM1_ESM.zip › Additional file 1/Additional file 1 Fig. S1.pdf]

**A**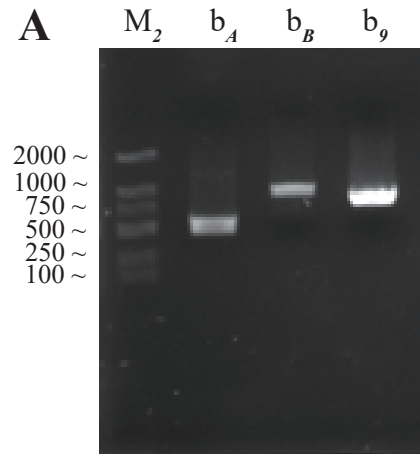**B**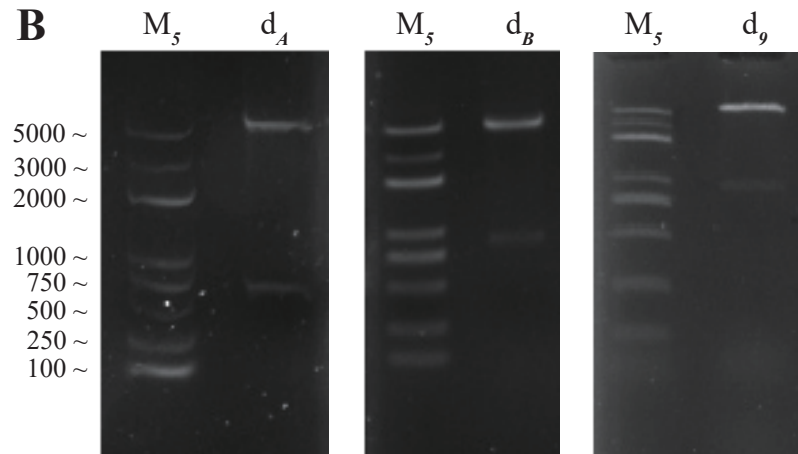

Supplement: Supplementary file 1 — Additional file 1: Fig. S1. Lipid metabolism and the predicted regulatory mechanisms in Corynebacterium glutamicum. There was a type I fatty acid synthase (FAS-I) system in C glutamicum, and the metabolites were CoA derivatives. As a TetR-type transcriptional regulator, FasR affected the transcriptional expression of genes including accD1, fasA, and fasB. Meanwhile, acyl-CoA could inhibit Acc, FasA, and FasB. This organism did not degrade fatty acid naturally due to the lack of the β-oxidation pathway. The red lines represented reference to previous studies (Ikeda et al. 2020), where double lines indicated inhibition and predicted inhibition and solid and dashed arrows indicated single and multiple enzymatic processes, respectively. The blue lines showed the predicted reverse β-oxidation pathway, a novel fatty acid synthesis pathway. Acetyl CoA carboxylase (Acc) was composed of AccBC, AccD1, and AccE. AccBC, acetyl-CoA carboxylase α subunit; AccD1, acetyl-CoA carboxylase β subunit; AccE, acyl carboxylase ε subunit; NCgl2309, acetyl-CoA acetyltransferase; NCgl0919, enoyl-CoA hydrolase; NCgl0973, acyl-CoA dehydrogenase; PD, pyruvate dehydrogenase; FasA, fatty acid synthase IA; FasB, fatty acid synthase IB; FasR, fatty acid synthesis inhibitory protein; Tes, acyl-CoA thioesterase; FadD, Acyl-CoA synthase. The dotted boxes in the figure were exogenous genes. FadE and Ter, acyl-CoA dehydrogenase; EchA, enoyl-CoA hydratase; FadB, multifunctional enoyl-CoA hydratase, 3-hydroxyacyl-CoA dehydrogenase; FadA, ketoacyl-CoA reductase. Fig S2. Agarose gel electrophoresis of the positive clones for heterologous expression in E. coli. A PCR validation of bacterial liquid. Lane M2, 2000 bp DNA marker; Lane bA, bacterial liquid PCR of BLtesA; Lane bB, bacterial liquid PCR of BLtesB; Lane b9, bacterial liquid PCR of BLte9; B double digestion validation of the recombinant plasmid. Lane M5, 5000 bp DNA marker; Lane dA, double digestion of plasmid pET_tesA extracted from BLtesA; Lane dB, [file 12934_2023_2189_MOESM1_ESM.zip › Additional file 1/Additional file 1 Fig. S2.pdf]

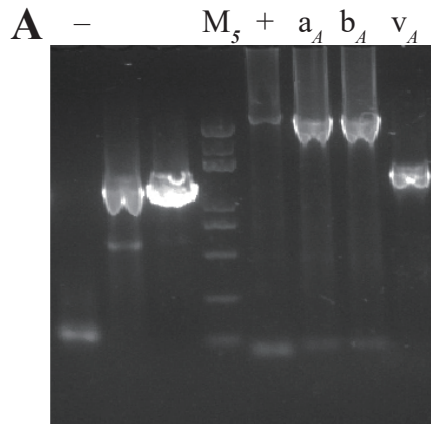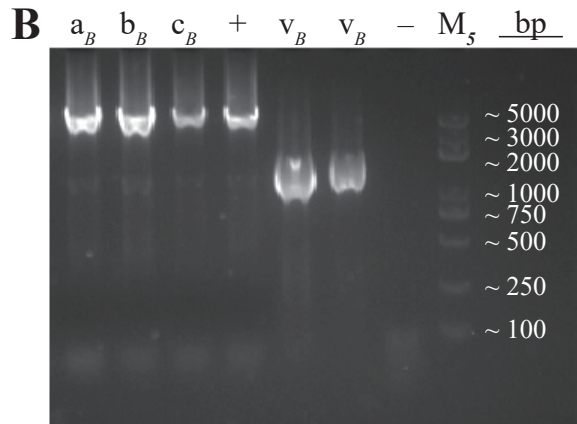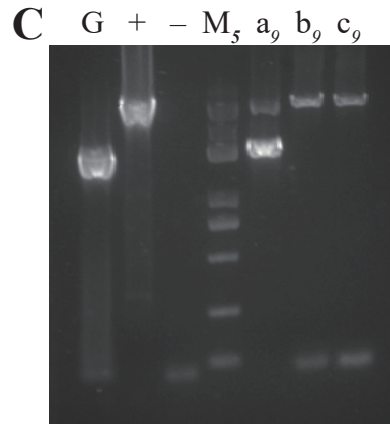

Supplement: Supplementary file 1 — Additional file 1: Fig. S1. Lipid metabolism and the predicted regulatory mechanisms in Corynebacterium glutamicum. There was a type I fatty acid synthase (FAS-I) system in C glutamicum, and the metabolites were CoA derivatives. As a TetR-type transcriptional regulator, FasR affected the transcriptional expression of genes including accD1, fasA, and fasB. Meanwhile, acyl-CoA could inhibit Acc, FasA, and FasB. This organism did not degrade fatty acid naturally due to the lack of the β-oxidation pathway. The red lines represented reference to previous studies (Ikeda et al. 2020), where double lines indicated inhibition and predicted inhibition and solid and dashed arrows indicated single and multiple enzymatic processes, respectively. The blue lines showed the predicted reverse β-oxidation pathway, a novel fatty acid synthesis pathway. Acetyl CoA carboxylase (Acc) was composed of AccBC, AccD1, and AccE. AccBC, acetyl-CoA carboxylase α subunit; AccD1, acetyl-CoA carboxylase β subunit; AccE, acyl carboxylase ε subunit; NCgl2309, acetyl-CoA acetyltransferase; NCgl0919, enoyl-CoA hydrolase; NCgl0973, acyl-CoA dehydrogenase; PD, pyruvate dehydrogenase; FasA, fatty acid synthase IA; FasB, fatty acid synthase IB; FasR, fatty acid synthesis inhibitory protein; Tes, acyl-CoA thioesterase; FadD, Acyl-CoA synthase. The dotted boxes in the figure were exogenous genes. FadE and Ter, acyl-CoA dehydrogenase; EchA, enoyl-CoA hydratase; FadB, multifunctional enoyl-CoA hydratase, 3-hydroxyacyl-CoA dehydrogenase; FadA, ketoacyl-CoA reductase. Fig S2. Agarose gel electrophoresis of the positive clones for heterologous expression in E. coli. A PCR validation of bacterial liquid. Lane M2, 2000 bp DNA marker; Lane bA, bacterial liquid PCR of BLtesA; Lane bB, bacterial liquid PCR of BLtesB; Lane b9, bacterial liquid PCR of BLte9; B double digestion validation of the recombinant plasmid. Lane M5, 5000 bp DNA marker; Lane dA, double digestion of plasmid pET_tesA extracted from BLtesA; Lane dB, [file 12934_2023_2189_MOESM1_ESM.zip › Additional file 1/Additional file 1 Fig. S3.pdf]

**A** $M_2$  $h_A$  $h_B$  $h_9$ 

2000 ~  
1000 ~  
750 ~  
500 ~  
250 ~  
100 ~

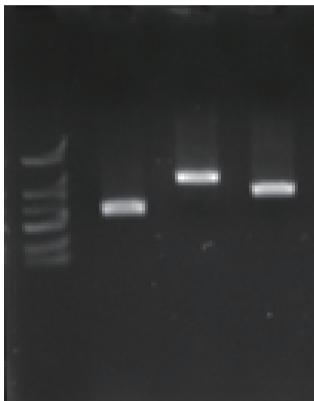**B** $M_5$  $q_A$  $q_B$  $q_9$ 

5000 ~  
3000 ~  
2000 ~  
1000 ~  
750 ~  
500 ~  
250 ~  
100 ~

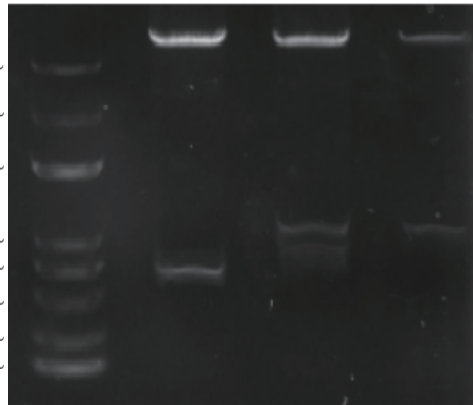

Supplement: Supplementary file 1 — Additional file 1: Fig. S1. Lipid metabolism and the predicted regulatory mechanisms in Corynebacterium glutamicum. There was a type I fatty acid synthase (FAS-I) system in C glutamicum, and the metabolites were CoA derivatives. As a TetR-type transcriptional regulator, FasR affected the transcriptional expression of genes including accD1, fasA, and fasB. Meanwhile, acyl-CoA could inhibit Acc, FasA, and FasB. This organism did not degrade fatty acid naturally due to the lack of the β-oxidation pathway. The red lines represented reference to previous studies (Ikeda et al. 2020), where double lines indicated inhibition and predicted inhibition and solid and dashed arrows indicated single and multiple enzymatic processes, respectively. The blue lines showed the predicted reverse β-oxidation pathway, a novel fatty acid synthesis pathway. Acetyl CoA carboxylase (Acc) was composed of AccBC, AccD1, and AccE. AccBC, acetyl-CoA carboxylase α subunit; AccD1, acetyl-CoA carboxylase β subunit; AccE, acyl carboxylase ε subunit; NCgl2309, acetyl-CoA acetyltransferase; NCgl0919, enoyl-CoA hydrolase; NCgl0973, acyl-CoA dehydrogenase; PD, pyruvate dehydrogenase; FasA, fatty acid synthase IA; FasB, fatty acid synthase IB; FasR, fatty acid synthesis inhibitory protein; Tes, acyl-CoA thioesterase; FadD, Acyl-CoA synthase. The dotted boxes in the figure were exogenous genes. FadE and Ter, acyl-CoA dehydrogenase; EchA, enoyl-CoA hydratase; FadB, multifunctional enoyl-CoA hydratase, 3-hydroxyacyl-CoA dehydrogenase; FadA, ketoacyl-CoA reductase. Fig S2. Agarose gel electrophoresis of the positive clones for heterologous expression in E. coli. A PCR validation of bacterial liquid. Lane M2, 2000 bp DNA marker; Lane bA, bacterial liquid PCR of BLtesA; Lane bB, bacterial liquid PCR of BLtesB; Lane b9, bacterial liquid PCR of BLte9; B double digestion validation of the recombinant plasmid. Lane M5, 5000 bp DNA marker; Lane dA, double digestion of plasmid pET_tesA extracted from BLtesA; Lane dB, [file 12934_2023_2189_MOESM1_ESM.zip › Additional file 1/Additional file 1 Fig. S4.pdf]

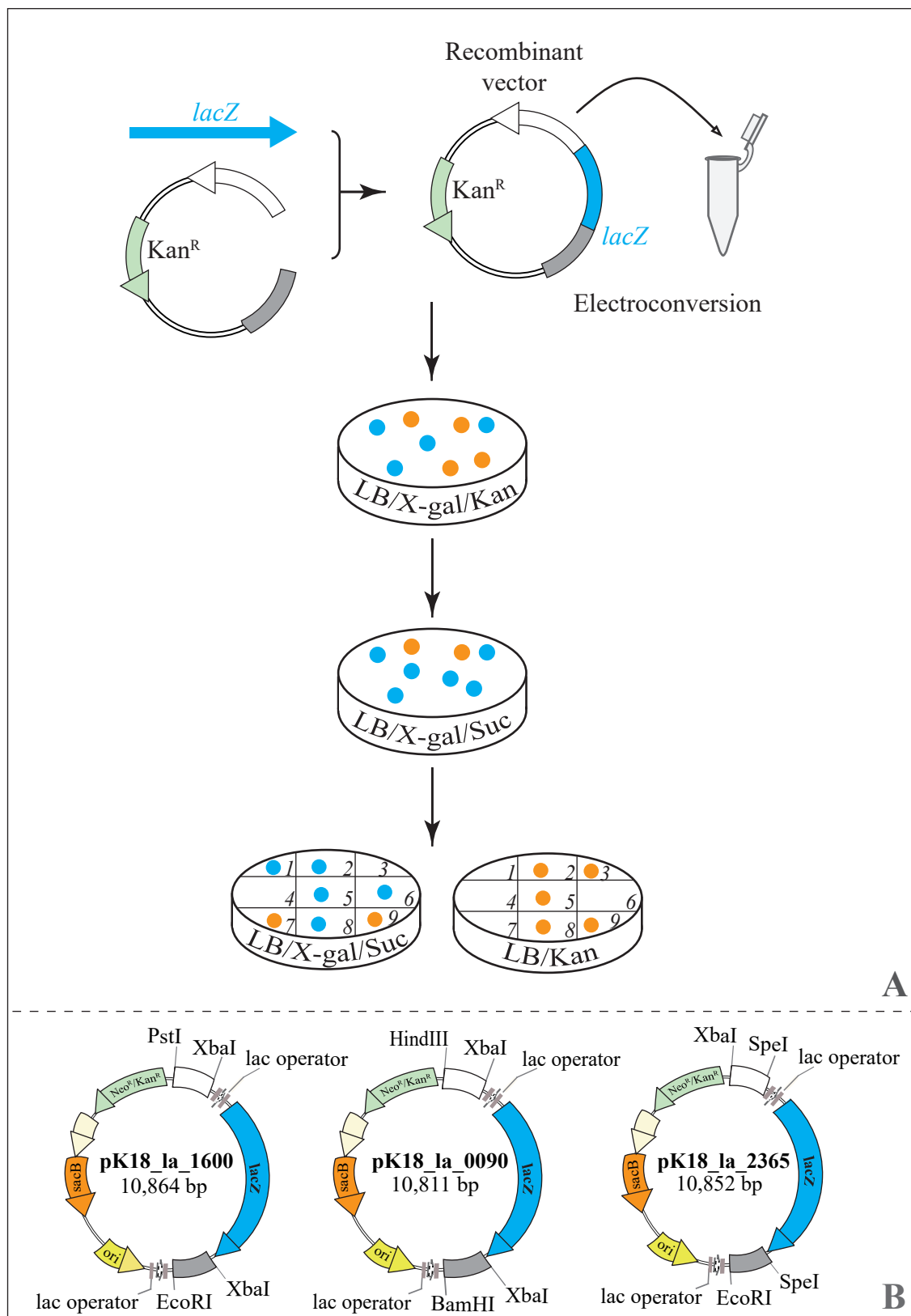

Supplement: Supplementary file 1 — Additional file 1: Fig. S1. Lipid metabolism and the predicted regulatory mechanisms in Corynebacterium glutamicum. There was a type I fatty acid synthase (FAS-I) system in C glutamicum, and the metabolites were CoA derivatives. As a TetR-type transcriptional regulator, FasR affected the transcriptional expression of genes including accD1, fasA, and fasB. Meanwhile, acyl-CoA could inhibit Acc, FasA, and FasB. This organism did not degrade fatty acid naturally due to the lack of the β-oxidation pathway. The red lines represented reference to previous studies (Ikeda et al. 2020), where double lines indicated inhibition and predicted inhibition and solid and dashed arrows indicated single and multiple enzymatic processes, respectively. The blue lines showed the predicted reverse β-oxidation pathway, a novel fatty acid synthesis pathway. Acetyl CoA carboxylase (Acc) was composed of AccBC, AccD1, and AccE. AccBC, acetyl-CoA carboxylase α subunit; AccD1, acetyl-CoA carboxylase β subunit; AccE, acyl carboxylase ε subunit; NCgl2309, acetyl-CoA acetyltransferase; NCgl0919, enoyl-CoA hydrolase; NCgl0973, acyl-CoA dehydrogenase; PD, pyruvate dehydrogenase; FasA, fatty acid synthase IA; FasB, fatty acid synthase IB; FasR, fatty acid synthesis inhibitory protein; Tes, acyl-CoA thioesterase; FadD, Acyl-CoA synthase. The dotted boxes in the figure were exogenous genes. FadE and Ter, acyl-CoA dehydrogenase; EchA, enoyl-CoA hydratase; FadB, multifunctional enoyl-CoA hydratase, 3-hydroxyacyl-CoA dehydrogenase; FadA, ketoacyl-CoA reductase. Fig S2. Agarose gel electrophoresis of the positive clones for heterologous expression in E. coli. A PCR validation of bacterial liquid. Lane M2, 2000 bp DNA marker; Lane bA, bacterial liquid PCR of BLtesA; Lane bB, bacterial liquid PCR of BLtesB; Lane b9, bacterial liquid PCR of BLte9; B double digestion validation of the recombinant plasmid. Lane M5, 5000 bp DNA marker; Lane dA, double digestion of plasmid pET_tesA extracted from BLtesA; Lane dB, [file 12934_2023_2189_MOESM1_ESM.zip › Additional file 1/Additional file 1 Fig. S5.pdf]

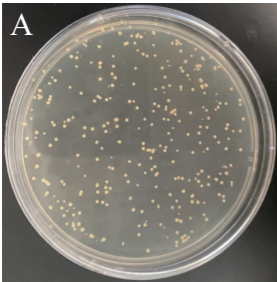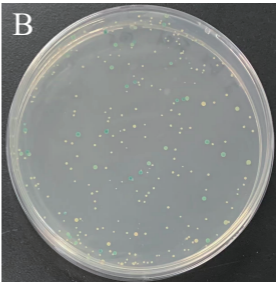

Supplement: Supplementary file 1 — Additional file 1: Fig. S1. Lipid metabolism and the predicted regulatory mechanisms in Corynebacterium glutamicum. There was a type I fatty acid synthase (FAS-I) system in C glutamicum, and the metabolites were CoA derivatives. As a TetR-type transcriptional regulator, FasR affected the transcriptional expression of genes including accD1, fasA, and fasB. Meanwhile, acyl-CoA could inhibit Acc, FasA, and FasB. This organism did not degrade fatty acid naturally due to the lack of the β-oxidation pathway. The red lines represented reference to previous studies (Ikeda et al. 2020), where double lines indicated inhibition and predicted inhibition and solid and dashed arrows indicated single and multiple enzymatic processes, respectively. The blue lines showed the predicted reverse β-oxidation pathway, a novel fatty acid synthesis pathway. Acetyl CoA carboxylase (Acc) was composed of AccBC, AccD1, and AccE. AccBC, acetyl-CoA carboxylase α subunit; AccD1, acetyl-CoA carboxylase β subunit; AccE, acyl carboxylase ε subunit; NCgl2309, acetyl-CoA acetyltransferase; NCgl0919, enoyl-CoA hydrolase; NCgl0973, acyl-CoA dehydrogenase; PD, pyruvate dehydrogenase; FasA, fatty acid synthase IA; FasB, fatty acid synthase IB; FasR, fatty acid synthesis inhibitory protein; Tes, acyl-CoA thioesterase; FadD, Acyl-CoA synthase. The dotted boxes in the figure were exogenous genes. FadE and Ter, acyl-CoA dehydrogenase; EchA, enoyl-CoA hydratase; FadB, multifunctional enoyl-CoA hydratase, 3-hydroxyacyl-CoA dehydrogenase; FadA, ketoacyl-CoA reductase. Fig S2. Agarose gel electrophoresis of the positive clones for heterologous expression in E. coli. A PCR validation of bacterial liquid. Lane M2, 2000 bp DNA marker; Lane bA, bacterial liquid PCR of BLtesA; Lane bB, bacterial liquid PCR of BLtesB; Lane b9, bacterial liquid PCR of BLte9; B double digestion validation of the recombinant plasmid. Lane M5, 5000 bp DNA marker; Lane dA, double digestion of plasmid pET_tesA extracted from BLtesA; Lane dB, [file 12934_2023_2189_MOESM1_ESM.zip › Additional file 1/Additional file 1 Fig. S6.pdf]

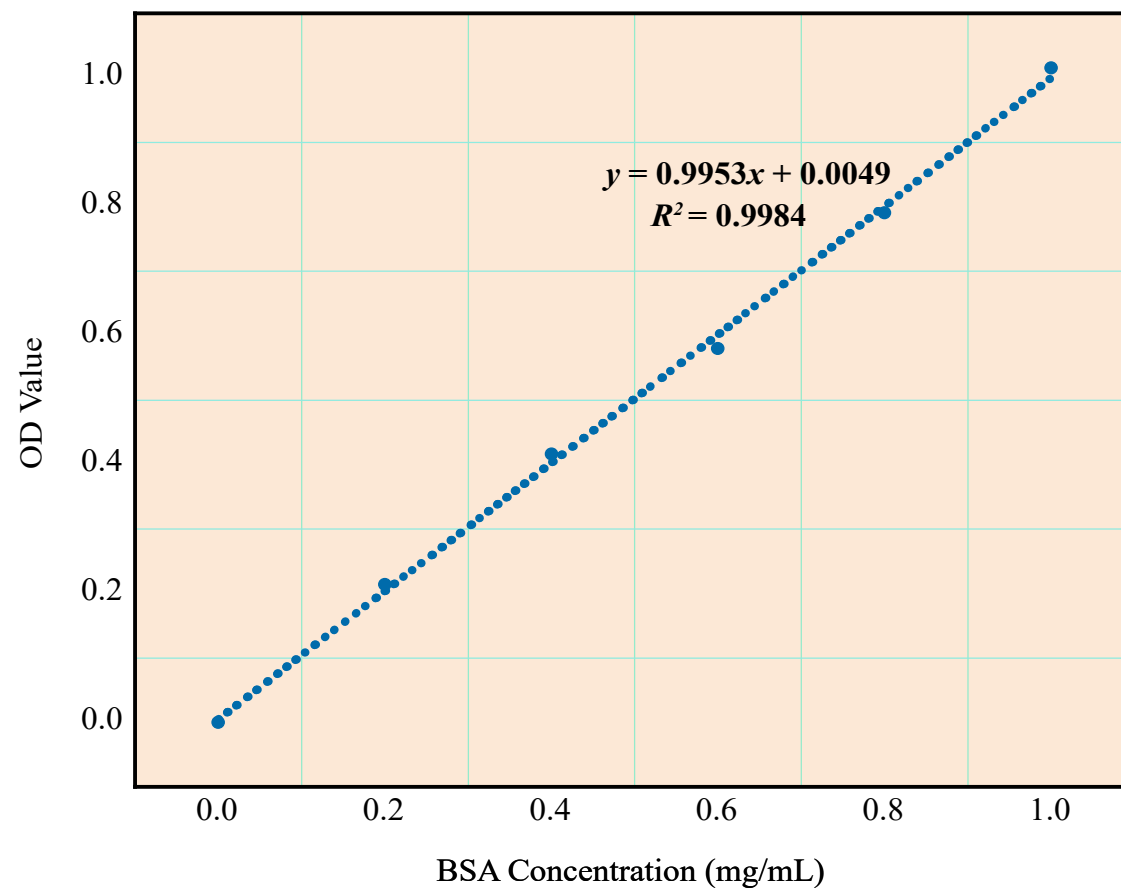

Supplement: Supplementary file 1 — Additional file 1: Fig. S1. Lipid metabolism and the predicted regulatory mechanisms in Corynebacterium glutamicum. There was a type I fatty acid synthase (FAS-I) system in C glutamicum, and the metabolites were CoA derivatives. As a TetR-type transcriptional regulator, FasR affected the transcriptional expression of genes including accD1, fasA, and fasB. Meanwhile, acyl-CoA could inhibit Acc, FasA, and FasB. This organism did not degrade fatty acid naturally due to the lack of the β-oxidation pathway. The red lines represented reference to previous studies (Ikeda et al. 2020), where double lines indicated inhibition and predicted inhibition and solid and dashed arrows indicated single and multiple enzymatic processes, respectively. The blue lines showed the predicted reverse β-oxidation pathway, a novel fatty acid synthesis pathway. Acetyl CoA carboxylase (Acc) was composed of AccBC, AccD1, and AccE. AccBC, acetyl-CoA carboxylase α subunit; AccD1, acetyl-CoA carboxylase β subunit; AccE, acyl carboxylase ε subunit; NCgl2309, acetyl-CoA acetyltransferase; NCgl0919, enoyl-CoA hydrolase; NCgl0973, acyl-CoA dehydrogenase; PD, pyruvate dehydrogenase; FasA, fatty acid synthase IA; FasB, fatty acid synthase IB; FasR, fatty acid synthesis inhibitory protein; Tes, acyl-CoA thioesterase; FadD, Acyl-CoA synthase. The dotted boxes in the figure were exogenous genes. FadE and Ter, acyl-CoA dehydrogenase; EchA, enoyl-CoA hydratase; FadB, multifunctional enoyl-CoA hydratase, 3-hydroxyacyl-CoA dehydrogenase; FadA, ketoacyl-CoA reductase. Fig S2. Agarose gel electrophoresis of the positive clones for heterologous expression in E. coli. A PCR validation of bacterial liquid. Lane M2, 2000 bp DNA marker; Lane bA, bacterial liquid PCR of BLtesA; Lane bB, bacterial liquid PCR of BLtesB; Lane b9, bacterial liquid PCR of BLte9; B double digestion validation of the recombinant plasmid. Lane M5, 5000 bp DNA marker; Lane dA, double digestion of plasmid pET_tesA extracted from BLtesA; Lane dB, [file 12934_2023_2189_MOESM1_ESM.zip › Additional file 1/Additional file 1 Fig. S7.pdf]

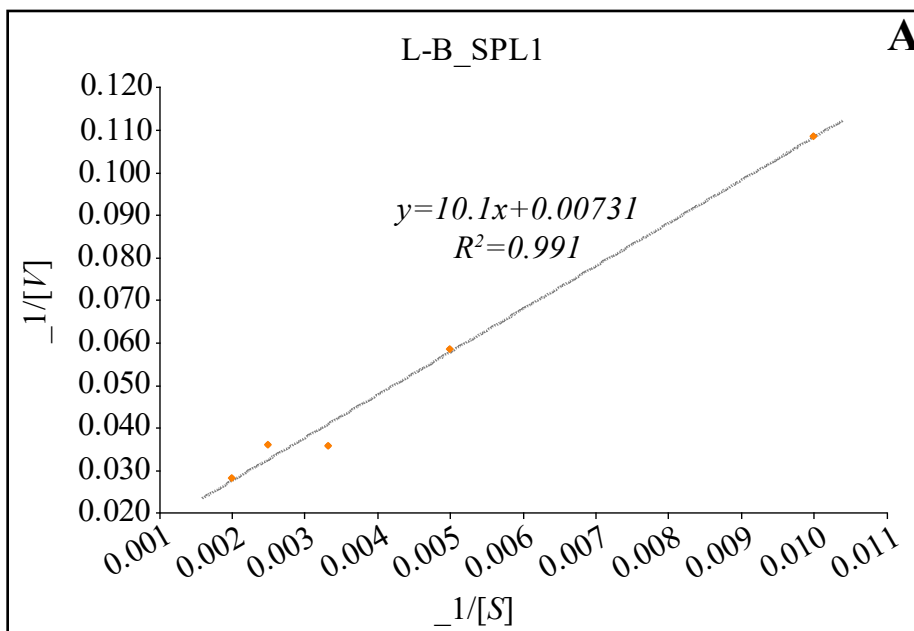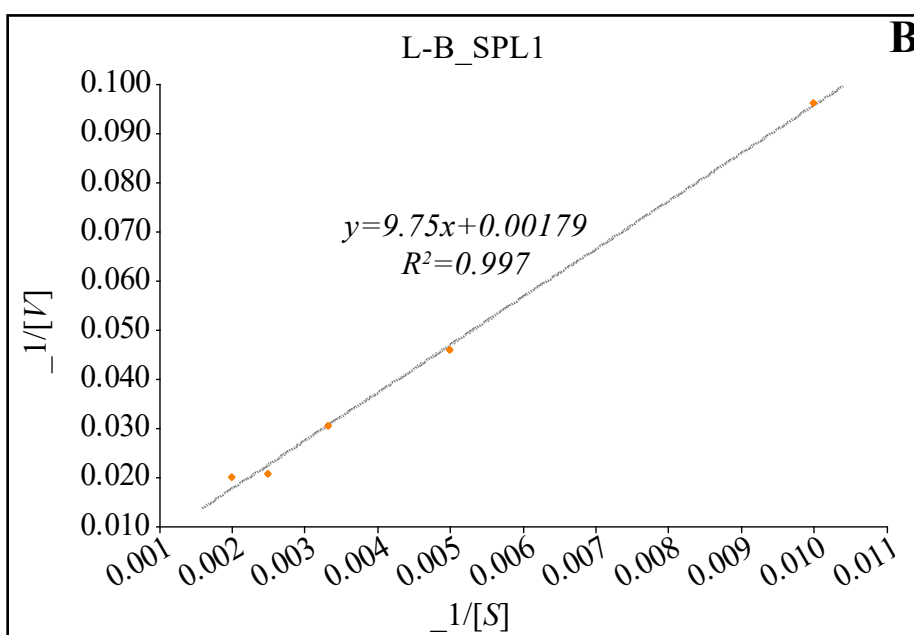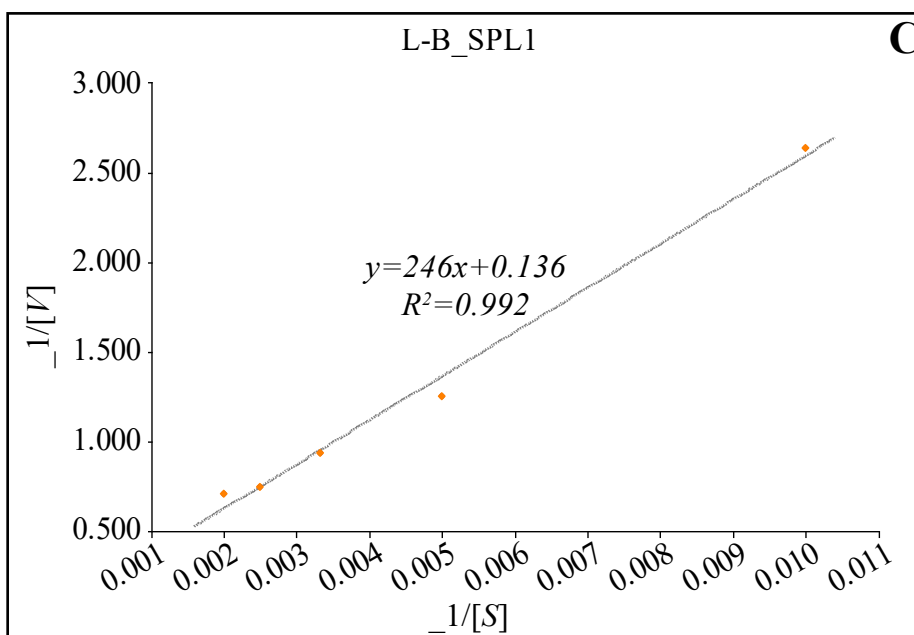

Supplement: Supplementary file 1 — Additional file 1: Fig. S1. Lipid metabolism and the predicted regulatory mechanisms in Corynebacterium glutamicum. There was a type I fatty acid synthase (FAS-I) system in C glutamicum, and the metabolites were CoA derivatives. As a TetR-type transcriptional regulator, FasR affected the transcriptional expression of genes including accD1, fasA, and fasB. Meanwhile, acyl-CoA could inhibit Acc, FasA, and FasB. This organism did not degrade fatty acid naturally due to the lack of the β-oxidation pathway. The red lines represented reference to previous studies (Ikeda et al. 2020), where double lines indicated inhibition and predicted inhibition and solid and dashed arrows indicated single and multiple enzymatic processes, respectively. The blue lines showed the predicted reverse β-oxidation pathway, a novel fatty acid synthesis pathway. Acetyl CoA carboxylase (Acc) was composed of AccBC, AccD1, and AccE. AccBC, acetyl-CoA carboxylase α subunit; AccD1, acetyl-CoA carboxylase β subunit; AccE, acyl carboxylase ε subunit; NCgl2309, acetyl-CoA acetyltransferase; NCgl0919, enoyl-CoA hydrolase; NCgl0973, acyl-CoA dehydrogenase; PD, pyruvate dehydrogenase; FasA, fatty acid synthase IA; FasB, fatty acid synthase IB; FasR, fatty acid synthesis inhibitory protein; Tes, acyl-CoA thioesterase; FadD, Acyl-CoA synthase. The dotted boxes in the figure were exogenous genes. FadE and Ter, acyl-CoA dehydrogenase; EchA, enoyl-CoA hydratase; FadB, multifunctional enoyl-CoA hydratase, 3-hydroxyacyl-CoA dehydrogenase; FadA, ketoacyl-CoA reductase. Fig S2. Agarose gel electrophoresis of the positive clones for heterologous expression in E. coli. A PCR validation of bacterial liquid. Lane M2, 2000 bp DNA marker; Lane bA, bacterial liquid PCR of BLtesA; Lane bB, bacterial liquid PCR of BLtesB; Lane b9, bacterial liquid PCR of BLte9; B double digestion validation of the recombinant plasmid. Lane M5, 5000 bp DNA marker; Lane dA, double digestion of plasmid pET_tesA extracted from BLtesA; Lane dB, [file 12934_2023_2189_MOESM1_ESM.zip › Additional file 1/Additional file 1 Fig. S8.pdf]
